# Screening and Characterizing Tyrosinase Inhibitors from *Salvia miltiorrhiza* and *Carthamus tinctorius* by Spectrum-Effect Relationship Analysis and Molecular Docking

**DOI:** 10.1155/2018/2141389

**Published:** 2018-05-09

**Authors:** Ya-Li Wang, Guang Hu, Qian Zhang, Yu-Xiu Yang, Qiao-Qiao Li, Yuan-Jia Hu, Hua Chen, Feng-Qing Yang

**Affiliations:** ^1^School of Pharmacy and Bioengineering, Chongqing University of Technology, Chongqing 400054, China; ^2^School of Chemistry and Chemical Engineering, Chongqing University, Chongqing 401331, China; ^3^State Key Laboratory of Quality Research in Chinese Medicine, Institute of Chinese Medical Sciences, University of Macau, Macau

## Abstract

Tyrosinase (TYR) is a rate-limiting enzyme in the synthesis of melanin, while direct TYR inhibitors are a class of important clinical antimelanoma drugs. This study established a spectrum-effect relationship analysis method and high-performance liquid chromatography-mass spectrometry (LC-MS) analysis method to screen and identify the active ingredients that inhibited TYR in *Salvia miltiorrhiza*–*Carthamus tinctorius* (Danshen–Honghua, DH) herbal pair. Seventeen potential active compounds (peaks) in the extract of DH herbal pair were predicted, and thirteen of them were tentatively identified by LC-MS analysis. Furthermore, TYR inhibitory activities of five pure compounds obtained from the DH herbal pair were validated in the test in which kojic acid served as a positive control drug. Among them, three compounds including protocatechuic aldehyde, hydroxysafflor yellow A, and tanshinone IIA were verified to have high TYR inhibitory activity (IC50 value of 455, 498, and 1214 *μ*M, resp.) and bind to the same amino acid residues in TYR catalytic pocket according to the results of the molecular docking test. However, the other two compounds lithospermic acid and salvianolic acid A had a weak effect on TYR, as they do not combine with the active amino acid residues or act on the active center of TYR. Therefore, the developed methods (spectrum-effect relationship analysis and molecular docking) could be used to effectively screen TYR inhibitors in complex mixtures such as natural products.

## 1. Introduction

Tyrosinase (TYR) belongs to the type 3 copper protein family containing dinuclear copper ions and widely exists in nature from microorganisms to humans [1]. It is a critical enzyme in the synthesis process of melanin pigments, catalyzing orthohydroxylation of monophenols to *o*-diphenols and then to the corresponding *o*-quinones [2]. However, overproduction of melanin pigments becomes a problem in the cosmetic and clinical points of view, such as melasma, freckles, and melanosis [3, 4]. Due to the importance of TYR during the synthesis of melanin, blocking the activity of TYR is one of the ideal strategies to treat melanin pigment diseases currently. To date, arbutin and kojic acid are the most commonly used tyrosinase inhibitors, which often serve as positive control drugs [5, 6]. However, traditional synthetic or microbial origin tyrosine inhibitors have some drawbacks, such as long-term contact of hydroquinone can lead to skin cancer, dermatitis, and other diseases [7, 8]. In reality, it is reported that the polyphenols and flavonoids isolated from natural plants have significant tyrosinase inhibition effect and less potential side effects [9, 10]. From this point of view, it is of great importance to screen TYR inhibitors from natural products.

Salviae Miltiorrhizae Radix et Rhizoma (Danshen in Chinese, DS) is the dried root or rhizome of *Salvia miltiorrhiza* Bunge, in which the main components are phenolic acids and diterpenes [11]. *Carthami flos* (Honghua in Chinese, HH), the dried flower of *Carthamus tinctorius* L., is generally composed of flavonoids, fatty acids, volatile oils, and polysaccharides [12]. In previous reports, the inhibitory effect of *Salvia miltiorrhiza* and *Carthamus tinctorius* on tyrosinase has been validated [13–15]; however, the active constituents with tyrosinase inhibition activity have not been clearly reported yet. Therefore, in this study, a spectrum-effect analysis method is developed to screen the active constituents that inhibit tyrosinase in Danshen–Honghua (DH) herbal pair. The spectrum-effect relationship analysis combines the chemical compositions of the fingerprint of natural products with the results of the efficacy, and is originally used to develop control standards that can truly reflect the inherent quality of products [16]. Furthermore, spectrum-effect analysis is also used to screen the active components from natural products [17]. In reality, spectrum-effect analysis shows some positive features such as reliability, time-saving capacity, and simple operation [18, 19].

In this study, the inhibition effect of DH herbal pair and single drug on tyrosinase was compared first. Then, the components in the DH herbal pair are analyzed and identified by HPLC analysis. Third, the active components in DH herbal pair were predicted by spectrum-effect analysis, and their structures were identified by LC-MS analysis. Furthermore, the TYR inhibition activities of the predicted compounds were evaluated in an *in vitro* model. Finally, molecular docking, which is a method of drug design through the characterization of the receptor and the interaction between the receptor and the drug molecules, and binding mode and affinity prediction [20], was used to confirm the binding sites of compounds with tyrosinase and to predict several possible TYR inhibitors which possess similar structure to the screened active compounds by molecular docking.

## 2. Materials and Methods

### 2.1. Chemicals and Materials

Tyrosinase (MW 128 kDa) from *Agaricus bisporus*, kojic acid (≥98%), and L-tyrosine were purchased from Sigma-Aldrich (St. Louis, MO, USA). Tyrosinase, kojic acid, and L-tyrosine were dissolved in 50 mM sodium phosphate buffer (pH 6.8) before use. The reference compounds protocatechuic aldehyde, hydroxysafflor yellow A, tanshinone IIA, lithospermic acid, and salvianolic acid A (≥98%, determined by HPLC) were obtained from PUSH Bio-Technology Co., Ltd. (Chengdu, China). HPLC-grade acetonitrile and formic acid were obtained from Beijing InnoChem Science & Technology Co., Ltd. (Beijing, China). All of the experimental water was purified by water purification system (ATSelem 1820A, Antesheng Environmental Protection Equipment Co., Ltd., Chongqing, China). All other chemicals and solvents, unless otherwise specified, were guaranteed reagent grade and purchased from Sigma-Aldrich Chemical Co. LLC. (St. Louis, MO, USA).

Crude drugs of Danshen and Honghua were both purchased from Chongqing Heping Pharmacy Co., Ltd. (Chongqing, China), in June 2017. The voucher specimens of *Salvia miltiorrhiza* Bunge (number SM2017090101) and *Carthamus tinctorius* L. (number CF2017090101) were deposited at the Pharmaceutical Engineering Laboratory in School of Chemistry and Chemical Engineering, Chongqing University, Chongqing, China.

### 2.2. Preparation of DH Extracts and Stock Solutions

All the dried raw DS and HH were pulverized and griddled through 50 mesh sieves (about 0.29 mm) prior to extraction. Seven different proportions of the herbs were prepared with ratios of 1 : 1, 2 : 1, 3 : 1, 5 : 1, 1 : 5, 1 : 3, and 1 : 2 (g/g) DS to HH, respectively. 20 g of DS and HH mixed powder was extracted with 200 mL water in a glass-stoppered conical flask at 75°C for 1.5 h. After extraction, the mixture was filtered through gauze, and the residue was collected and extracted with the above process for a second time. The two filtrates were combined and evaporated in a rotary evaporator (ZFQ 85A, Shanghai Medical Instrument Special Factory, Shanghai, China) at 55°C under reducing pressure to remove the solvent. The extracts were further dried by lyophilization with freezing-drying system (DZF-6050, Shanghai Jing Hong Laboratory Instrument Co., Ltd., Shanghai, China) to obtain the DH extracts at a yield of about 25% (w/w, dried extract/crude herb). All pre- and postdilution solutions were stored at 4°C. Before HPLC analysis, the sample solutions were filtered through a 0.22 *μ*m nylon membrane filter (Shanghai Titan Scientific Co., Ltd., Shanghai, China).

The reference substances protocatechuic aldehyde, hydroxysafflor yellow A, tanshinone IIA, lithospermic acid, and salvianolic acid A and a positive control (kojic acid) were all prepared by dissolving the respective substance in methanol solution and diluted with PBS (50 mM, pH 6.8) to the required concentrations for TYR inhibitory and binding assay, respectively. All the solutions were stored at 4°C in dark before use.

### 2.3. HPLC and LC-MS Analysis

HPLC analysis was performed on an Agilent 1260 series liquid chromatograph system (Agilent Technologies, Palo Alto, CA, USA), which was equipped with a vacuum degasser, a binary pump, an autosampler, and a diode array detector (DAD), controlled by Agilent ChemStation software. An Agilent Zorbax SB-Aq column (250 mm × 4.6 mm, 5 *μ*m) preceded by a Zorbax SB-C_18_ guard column (12.5 × 4.6 mm, 5 *μ*m) was adopted for the analysis. The mobile phase consisted of solvent A (0.1% formic acid aqueous solution) and solvent B (acetonitrile) using a gradient elution, which was programmed as follows: 5% B at 0–2 min, 5%–15% B at 2–10 min, 15%–22% B at 10–24 min, 22%–29% B at 24–35 min, 29%–37% B at 35–42 min, and 37%–5% B at 42–47 min. The mobile phase was set at a flow rate of 1.0 mL/min with 10 *μ*L per sample injection. The UV detection wavelength was set at 280 nm, and the column temperature was conditioned at 37°C.

Shimadzu LC/MS-MS 8060 electrospray ionization-mass spectrometer (ESI-MS), consisting of a Triple Quadruple Detector (TSQ) as the mass detector (Shimadzu, Kyoto, Japan) and coupled with HPLC, was used for LC-MS identification. The LC conditions were the same as described previously. The ESI-MS conditions were as follows: the ESI was used in both positive and negative mode; nitrogen gas was used for desolvation at a flow rate of 3 L/min at 250°C; the temperature and flow rate of drying gas were set under 400°C and 10 L/min, respectively; the cone voltage was (+) 20 and (−) 20 V; MS data were recorded in the full-scan mode (*m/z* 50–1500), and MS^2^ data were recorded in the range of *m/z* 50–1200.

### 2.4. TYR Inhibitory Activity Assay

The enzyme assay was performed in 96-well Corining Costar plates (Corning Incorporated, USA). 50 *μ*L test solution and 50 *μ*L TYR solution (800 U/mL) were mixed and incubated for 10 min at room temperature. After the incubation, 100 *μ*L (1 mg/mL) of L-tyrosine in PBS (pH 6.8) buffer as chromogenic substrate was added to start the reaction. The absorbance was monitored at 490 nm every 30 s for 10 min with an iMark™ Microplate Absorbance Reader (Bio-Rad Laboratories, Inc., USA). PBS (50 mM, pH 6.8) buffer was prepared as blank control, and kojic acid was used as positive control. TYR inhibition activity was expressed as the inhibitory percentage of TYR:

(1) $$\begin{array}{c} \text{Inhibition percentage }\left(\%\right)=\frac{{\left(dA/dt\right)}_{\text{blank}}-{\left(dA/dt\right)}_{\text{sample}}}{{\left(dA/dt\right)}_{\text{blank}}}\times 100\%, \end{array}$$

where(*dA*/*dt*)_blank_ and (*dA*/*dt*)_sample_ are the reaction rate of the blank and sample group, respectively. All trials were independently performed in triplicates, and the results were shown with mean value of the triplicate observations.

### 2.5. Spectrum-Effect Relationship Analysis

Spectrum-effect analysis was performed by transferring DH fingerprint peak area and TYR inhibition activity test results into SPSS software for canonical correlation analysis (CCA). The optimized HPLC fingerprints of seven ratios DH samples were calculated and generated by professional software named “Similarity Evaluation System for Chromatographic Fingerprint of Traditional Chinese Medicine” composed by Chinese Pharmacopoeia Committee (Version 2012). CCA was used to assess the spectrum-effect relationships between the areas of 86 peaks in fingerprint and the TYR inhibition ratios.

### 2.6. In Silico Molecular Docking of TYR and Identified Active Compounds

Auto Dock 4.2 program (The Scripps Research Institute, La Jolla, CA, USA) was employed for *in silico* molecular docking study to validate the binding potency of the compounds to TYR [21]. The docking operation was performed according to the following steps. First, the crystal structure file of TYR (*Agaricus bisporus* mushroom tyrosinase) complex (PDB ID = 2y9x) was downloaded [22]. The dimension grid box (90 Å × 90 Å × 102 Å) and the grid spacing of 0.619 Å were defined to enclose the active site. Second, the ligand was deleted using UCSF Chimera, and unnecessary water molecules were removed, and hydrogen atoms were added [23]. Third, the 3D chemical structure of investigated compounds was drawn by using Microsoft office 3D and output in PDB format with minimized energy.

With the aim of docking with Autodock Vina, the grid size was set to (*x*, *y*, *z*) = (50, 50, 50) and the grid center was set to (*x*, *y*, *z*) = (10.044, 28.706, 43.443). In each simulation process, progress with default parameters run from Autogrid and Autodock. Lamarckian genetic algorithm (LGA) was used to find the most favorable ligand binding orientations, and the number of LGA runs is equal to 50. The interaction figures were generated, and the results of docking were recorded with binding energies and bonded residues.

### 2.7. Statistical Analysis

All data are presented as mean ±standard deviations (SD) of at least three different experiments. The statistical analysis was performed with SPSS (version 24, SPSS, Inc., Chicago, IL, USA).

## 3. Results and Discussion

### 3.1. Effects of DH Extracts on Tyrosinase Activity

**Figure 1:**
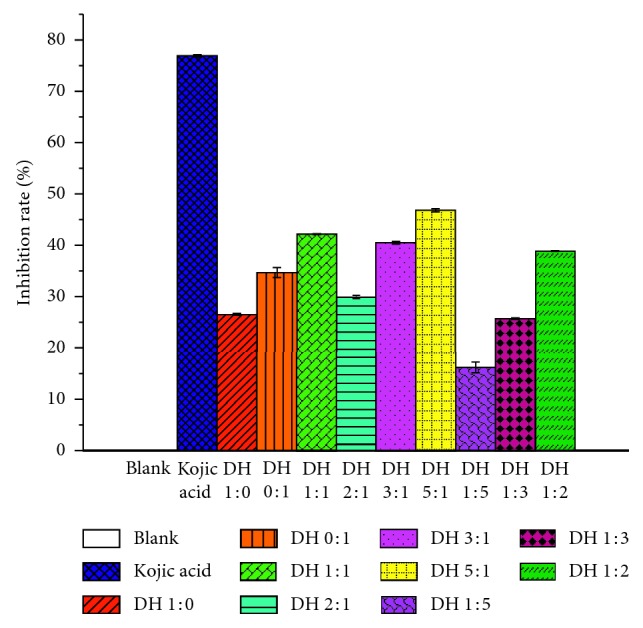
Different ratios of DH herbal pair on tyrosinase inhibition effects.

As shown in Table 1 and Figure 1, PBS served as a blank control, while kojic acid served as a positive control. Some DH herbal pair extracts (1 : 5 and 1 : 3) showed a weaker inhibitory effect than single herbal extracts when the concentrations of tyrosinase and the sample were kept constant. However, other DH herbal pair extracts (1 : 1, 3 : 1, 5 : 1, and 1 : 2) displayed a stronger inhibitory effect than single herbal extracts, which indicated that a synergistic effect of the herbal pair may occur on the inhibition of tyrosinase activity. Therefore, DH herbal pair was used as the research object for screening their tyrosinase inhibitors.

In order to obtain the best screening performance for active compounds in the complex matrix, some important parameters of the method including incubation time, TYR concentration, and sample concentration should be optimized. According to previous research, the incubation time might be controlled at the range of 30–120 min because a short incubation time (less than 30 min) might prevent the identification of target molecules which are not firmly bound to TYR, while a long incubation time (about 120 minutes) would not make significant influence on the screening result [24, 25]. After investigation, a proper TYR concentration (800 U/mL) increased the sensitivity and number of bioactive constituents detected in the sample; meanwhile, the inhibition effect of tyrosinase is most stable by the incubation time of 40 min (data not shown). Therefore, a sufficient incubation time (40 min) and a sufficient TYR concentration (800 U/mL) were used in this study.

### 3.2. Spectrum-Effect Relationship Analysis

**Figure 2:**
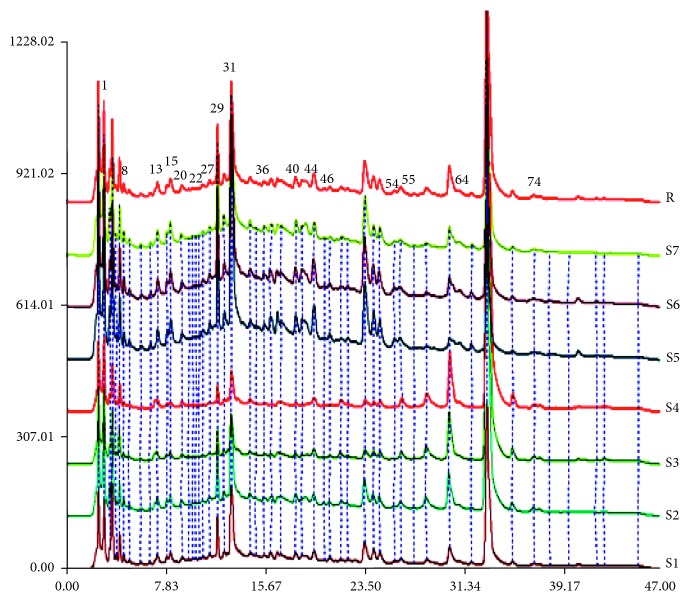
HPLC calibration fingerprints of DH herbal pair with different ratios. The chromatograms of S1–S7 are represented as follows: DH 1 : 1 (S1); DH 2 : 1 (S2); DH 3 : 1 (S3); DH 5 : 1 (S4); DH 1 : 5 (S5); DH 1 : 3 (S6); DH 1 : 2 (S7); control map (R).

The optimized HPLC fingerprints of DH samples with seven ratios are shown in Figure 2. A total of 86 peaks involved were detected in the calculation of spectrum-effect relationship. CCA (canonical correlation analysis) was used to assess the spectrum-effect relationship between the areas of 86 peaks and the main parameters (inhibition rate), and the results are shown in Table 2. As suggested by the correlation coefficients, the highly relevant peaks were 1, 8, 13, 15, 20, 22, 27, 29, 31, 36, 40, 44, 46, 54, 55, 64, and 74 with Pearson relational grade more than 0.8. In other words, these 17 peaks might be the main active components of herbal pair for inhibiting tyrosinase, and further studies were performed to identify the structures of these peaks to confirm their bioactivities.

### 3.3. Identification of the Potential TYR-Targeted Compound by LC-MS Analysis

**Figure 3:**
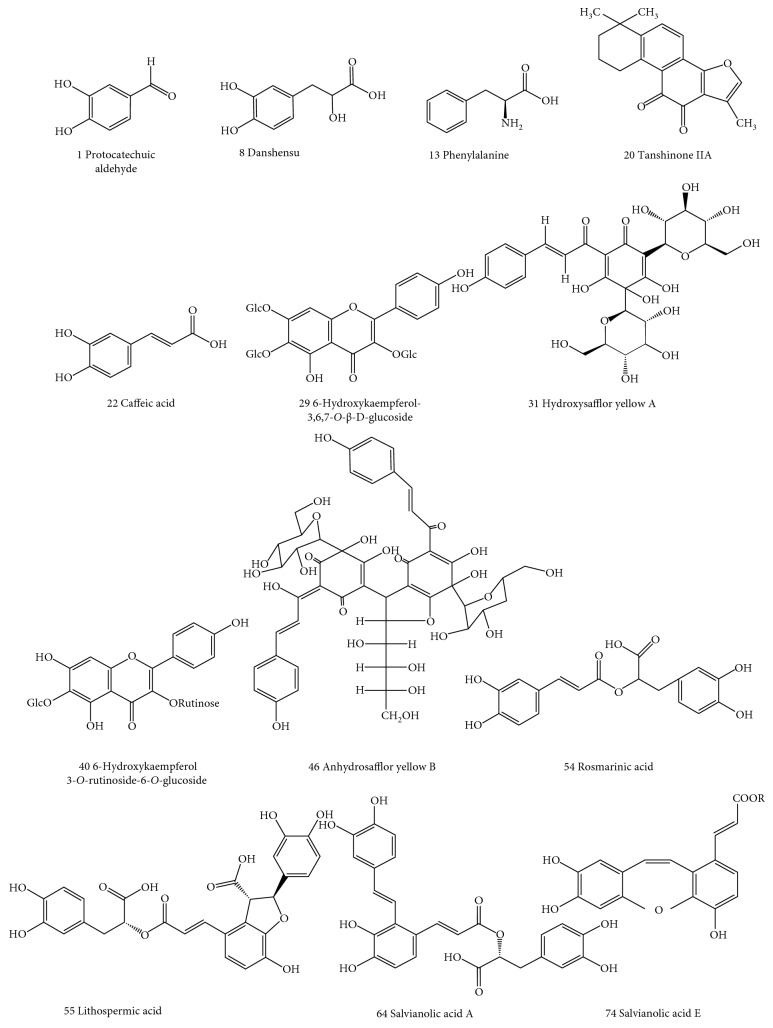
Chemical structures of compounds identified in DH herbal pair. The numbers of compounds are the same as the peak numbers in Figure 2.

HPLC-MS/MS analysis was used to identify the chemical structures of compounds in DH extracts. Based on the fragmentation behaviors, retention time and MS data (Table 3) of the peaks in the test samples, 13 compounds (protocatechuic aldehyde, danshensu, phenylalanine, tanshinone IIA, caffeic acid, 6-hydroxykaempferol-3,6,7-*O*-*β*-D-glucoside, hydroxysafflor yellow A, rosmarinic acid, anhydrosafflor yellow B, 6-hydroxykaempferol 3-*O*-rutinoside-6-*O*-glucoside, lithospermic acid, salvianolic acid A, and salvianolic acid E) were tentatively identified, and the structures of these compounds are shown in Figure 3. Five pure reference compounds including protocatechuic aldehyde, tanshinone IIA, hydroxysafflor yellow A, lithospermic acid, and salvianolic acid A were obtained for further *in vitro* activity tests.

### 3.4. In Vitro Activity Tests for the Predicted Compounds

**Figure 4:**
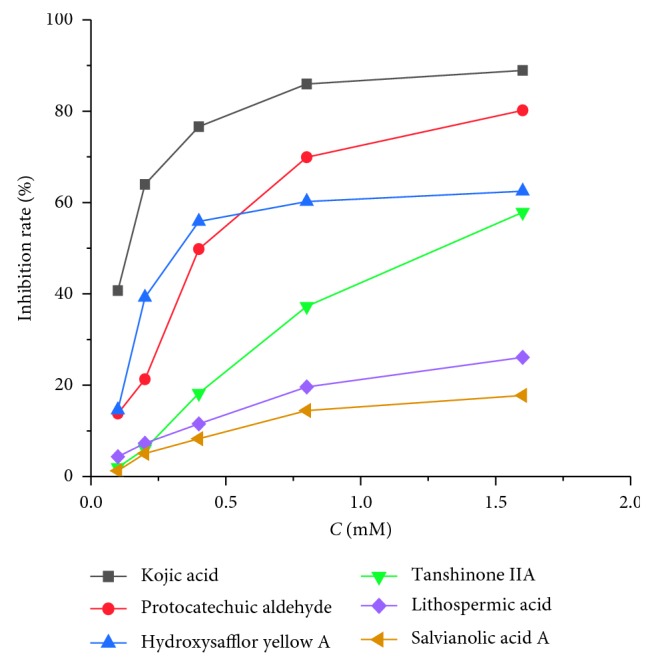
Inhibitory rate of active components to tyrosinase with different concentrations.

To confirm the ability of the hit compounds with TYR inhibitory activity, *in vitro* enzymatic activity assays were performed. Five concentrations of each compound were tested, and the results are shown in Table 4. As a well-known TYR inhibitor [5], kojic acid showed strong inhibition effect with a IC50 value of 127 *μ*M. From the results shown in Figure 4, among the five identified hit compounds, protocatechuic aldehyde, hydroxysafflor yellow A, and tanshinone IIA possessed strong TYR inhibition effects in a dose-dependent manner, with the IC50 values of 455, 498, and 1214 *μ*M, respectively. However, lithospermic acid and salvianolic acid A did not show significant inhibitory effect on tyrosinase at a relatively high concentration (1.6 mM). The reason may be that they do not combine with the active amino acid residues or do not act on the active center of TYR, but further studies are required.

### 3.5. Molecular Docking of TYR and Identified Active Compounds

**Figure 5:**
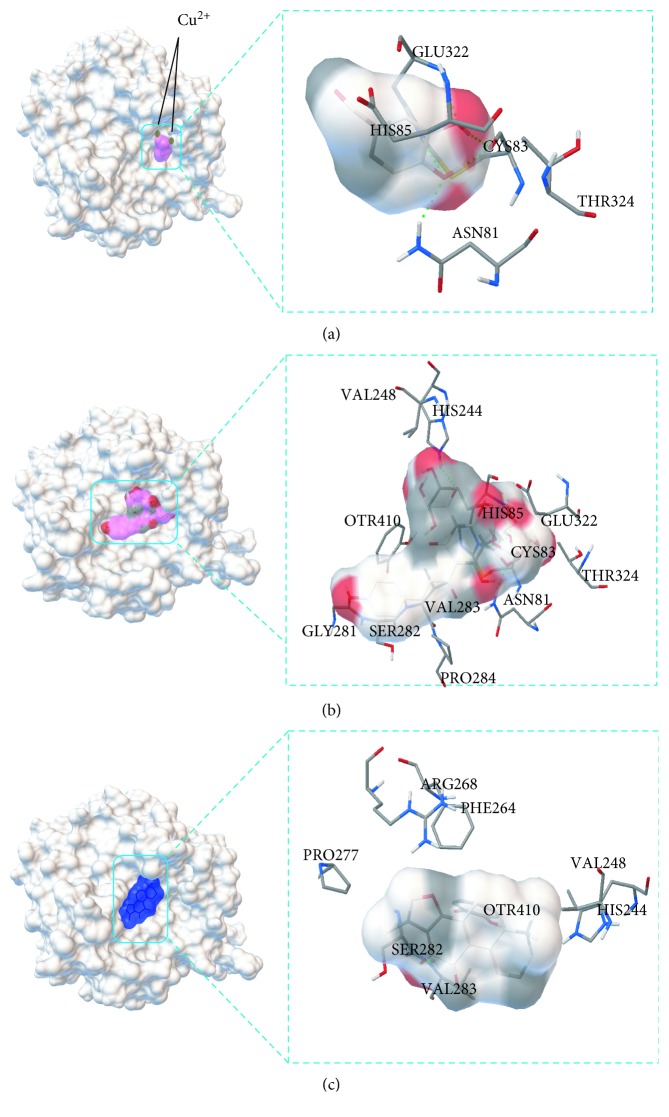
Molecular docking results of protocatechuic aldehyde (a), hydroxysafflor yellow A (b), and tanshinone IIA (c) with tyrosinase.

Molecular docking can be used to study the binding mechanism of compounds interacting with proteins. In this study, Autodock 4.2 was selected as the docking software to check out the active site of those components screened from DH extracts combined with TYR. The crystal structure of tyrosinase includes four identical parts, and one of them was used as the crystal structure of tyrosinase for computational docking analysis. Interestingly, as shown in Figures 5(a) and 5(b), protocatechuic aldehyde and hydroxysafflor yellow A could bond to the same catalytically active amino acid residues (THR324, ASN81, CYS83, GLU322, and HIS85) of TYR, which could explain the similar TYR inhibitory activity of these two compounds. Moreover, three hydrogen bonds (green line) between protocatechuic aldehyde, hydroxysafflor yellow A, and the amino acid residues were observed. As shown in Figure 5(c), tanshinone IIA was found bonding into the hydrophobic cavity of tyrosinase (blue region) and surrounded by amino acid residues VAL248, HIS244, OTR410, VAL283, SER282, PRO277, PHE264, and ARG268 of tyrosinase. The amino acid residues of the tyrosinase to which the active compound binds are shown in Table 5. The reason for the weak activity may be that lithospermic acid and salvianolic acid A do not combine with the active amino acid residues (such as GLU322 and THR324) or do not act on the active center (Cu^2+^) of tyrosinase. As tyrosinase is a copper-containing enzyme, it is expected that potential tyrosinase inhibitors should show high binding affinity for copper ions [26].

Along with the screening result, molecular docking was also carried out on compounds (absence of pure reference substances) having similar structure to protocatechuic aldehyde, lithospermic acid, and salvianolic acid A (the structure of these compounds are shown in Figure 4), attempting to explore other active TYR inhibitors by perspective of structure-activity relationship. As shown in Figure 5(a), the important region of protocatechuic aldehyde for copper chelation was the catechol structure. Compounds that functionally chelate copper ions at tyrosinase active sites have been frequently reported as effective tyrosinase inhibitors because they are analogous to the phenolic hydroxyl substrates of tyrosinase [27, 28]. In addition, danshensu, caffeic acid, rosmarinic acid, and salvianolic acid E having a catechol structure might also be potential TYR inhibitors. The docking result is shown in Table 5. With a portion of the same active sites with screened active compounds bound to TYR, it could be found that danshensu, caffeic acid, rosmarinic acid, salvianolic acid E, anhydrosafflor yellow B, 6-hydroxykaempferol-3,6,7-O-*β*-D-glucoside, and 6-hydroxykaempferol 3-*O*-rutinoside-6-*O*-glucoside similar to protocatechuic aldehyde, hydroxysafflor yellow A, and tanshinone IIA might also be potential TYR inhibitors. However, pure substances of these compounds were required for the further activity tests.

## 4. Conclusions

In this study, the TYR inhibitor screening methods were established, and the potential TYR inhibitory components from DH extract were screened. Combining the results of spectrum-effect analysis, LC-MS analysis, and enzymatic activity assay, three active compounds including protocatechuic aldehyde, hydroxysafflor yellow A, and tanshinone IIA were discovered as inhibitors targeting TYR. Meanwhile, docking results showed that these compounds might bind to the same amino acid residues in TYR catalytic pocket. Additionally, other potential active TYR inhibitors such as danshensu, caffeic acid, rosmarinic acid, and salvianolic acid E, which gained similar structures with the hit compounds, might also be identified. These results proved that the proposed method could effectively screen TYR inhibitors in complex mixtures and provided a reference for the discovery of other active TYR inhibitors.

## Figures and Tables

**Table 1:** Inhibition effect of DH extracts on tyrosinase with different ratios (*n* = 9).

Sample	Inhibition rate (%)
Blank^a^	0.06 ± 0.01
Kojic acid^b^	76.83 ± 0.24
DH 1 : 0^b^	26.55 ± 0.17
DH 0 : 1^b^	34.70 ± 0.98
DH 1 : 1^b^	42.19 ± 0.10
DH 2 : 1^b^	29.93 ± 0.32
DH 3 : 1^b^	40.53 ± 0.28
DH 5 : 1^b^	46.84 ± 0.30
DH 1 : 5^b^	16.15 ± 1.05
DH 1 : 3^b^	25.72 ± 0.17
DH 1 : 2^b^	38.90 ± 0.09

^a^Concentration: PBS (50 mM, pH6.8); ^b^concentration: 500 *μ*g/mL.

**Table 2:** Correlation coefficients between chromatogram peaks and inhibition rates.

Peak number	1	2	3	4	5	6	7	8	9	10	11	12	13
Inhibition rate	0.940^∗^	−0.702	−0.695	−0.501	−0.753	−0.769	−0.677	0.812^∗^	−0.721	−0.775	−0.790	−0.351	0.807^∗^
Peak number	**14**	**15**	**16**	**17**	**18**	**19**	**20**	**21**	**22**	**23**	**24**	**25**	**26**
Inhibition rate	0.510	−0.878^∗^	−0.742	−0.632	−0.713	0.714	0.919^∗^	−0.746	0.903^∗^	−0.129	−0.615	−0.233	−0.623
Peak number	27	28	29	30	31	32	33	34	35	36	37	38	39
Inhibition rate	0.952^∗^	−0.544	0.908^∗^	0.650	0.900^∗^	−0.397	−0.015	−0.466	0.321	−0.824^∗^	−0.795	−0.726	−0.762
Peak number	**40**	**41**	**42**	**43**	**44**	**45**	**46**	**47**	**48**	**49**	**50**	**51**	**52**
Inhibition rate	0.892^∗^	−0.571	−0.790	−0.785	−0.867^∗^	−0.745	0.891^∗^	−0.640	−0.746	−0.708	0.135	−0.753	−0.451
Peak number	**53**	**54**	**55**	**56**	**57**	**58**	**59**	**60**	**61**	**62**	**63**	**64**	**65**
Inhibition rate	−0.627	−0.943^∗^	−0.929^∗^	−0.759	−0.778	−0.037	−0.769	0.591	−0.621	−0.741	0.786	0.824^∗^	−0.725
Peak number	**66**	**67**	**68**	**69**	**70**	**71**	**72**	**73**	**74**	**75**	**76**	**77**	**78**
Inhibition rate	−0.667	−0.772	0.596	0.618	0.124	0.704	−0.712	0.654	0.887^∗^	0.215	0.361	0.371	0.718
Peak number	**79**	**80**	**81**	**82**	**83**	**84**	**85**	**86**					
Inhibition rate	0.610	0.646	−0.733	0.565	−0.717	0.279	−0.123	−0.512					

*Note*. Pearson correlation, “r” represents the relevant strength; ^∗^0.8 ≤ |*r*| ≤ 1 means Very significant correlation.

**Table 3:** HPLC-MS/MS data of 17 predicted active compounds from DH herbal pair.

Peak No.	*t*_R_ (min)	MW	MS^1^ (*m/z*)	MS^2^ (*m/z*)	Formula	Structural identification
**1**	3.087	138	138.05	92; 78; 65	C_7_H_6_O_3_	Protocatechuic aldehyde
**8**	3.112	198	197.05	178	C_9_H_10_O_5_	Danshensu
**13**	6.491	165	166.09	120	C_9_H_11_NO_2_	Phenylalanine
**15**	7.108	—	—	—	—	Unknown
**20**	7.952	294	295.15	277; 249	C_19_H_18_O_3_	Tanshinone IIA
**22**	8.951	180	179.10	135	C_9_H_8_O_4_	Caffeic acid
**27**	11.207	—	—	—	—	Unknown
**29**	12.386	788	787.40	625; 505; 463; 301	C_33_H_40_O_22_	6-Hydroxykaempferol-3,6,7-*O*-*β*-D-glucoside
**31**	12.771	612	611.30	491; 473; 403; 353; 325; 283; 205	C_27_H_32_O_16_	Hydroxysafflor yellow A
**36**	14.094	640	639.30	463; 362; 300; 255; 139	—	Unknown
**40**	17.091	772	773.35	695; 672; 303; 187; 112	C_33_H_40_O_21_	6-Hydroxykaempferol 3-*O*-rutinoside-6-*O*-glucoside
**44**	21.352	—	—	—	—	Unknown
**46**	23.969	1044	1043.45	1025; 923; 863; 764; 593; 449	C_48_H_52_O_26_	Anhydrosafflor yellow B
**54**	29.882	360	359.25	179; 161; 133	C_18_H_16_O_8_	Rosmarinic acid
**55**	31.813	538	537.25	295; 253; 203	C_27_H_22_O_12_	Lithospermic acid
**64**	32.528	494	493.25	295	C_26_H_22_O_10_	Salvianolic acid A
**74**	36.747	718	717.35	673; 617; 519; 321	—	Salvianolic acid E

**Table 4:** Inhibition effect of protocatechuic aldehyde, tanshinone IIA, hydroxysafflor yellow A, lithospermic acid, and salvianolic acid A on tyrosinase (*n*=9).

Compounds	Concentration (mmol)	Inhibition rate (%)
Blank	PBS (50 mM, pH 6.8)	0.44 ± 0.02

Kojic acid	1.6	88.93 ± 0.28
	0.8	85.97 ± 0.45
	0.4	76.63 ± 1.02
	0.2	63.97 ± 2.11
	0.1	40.72 ± 0.15

Protocatechuic aldehyde	1.6	80.18 ± 0.08
	0.8	69.91 ± 2.97
	0.4	49.80 ± 1.30
	0.2	21.29 ± 1.01
	0.1	13.78 ± 0.95

Tanshinone IIA	1.6	57.88 ± 1.98
	0.8	37.26 ± 2.87
	0.4	18.23 ± 2.23
	0.2	6.03 ± 1.12
	0.1	1.98 ± 0.05

Hydroxysafflor yellow A	1.6	62.48 ± 1.34
	0.8	60.22 ± 2.28
	0.4	55.85 ± 1.80
	0.2	39.23 ± 1.12
	0.1	14.58 ± 0.55

Lithospermic acid	1.6	26.09 ± 3.28
	0.8	19.62 ± 2.22
	0.4	11.52 ± 2.64
	0.2	7.28 ± 1.01
	0.1	4.32 ± 0.21

Salvianolic acid A	1.6	17.74 ± 3.06
	0.8	14.42 ± 1.14
	0.4	8.25 ± 1.98
	0.2	5.04 ± 0.91
	0.1	1.25 ± 1.32

**Table 5:** Binding residues of identified compounds in DH herbal pair with tyrosinase.

Compound	Residues	Residues with hydrogen bonding
Kojic acid	ASN81, GLU322, CYS83, THR324, THR84, and HIS85	ASN81, CYS83, and HIS85

Protocatechuic aldehyde	THR324, ASN81, CYS83, GLU322, and HIS85	ASN81, CYS83, and HIS85

Hydroxysafflor yellow A	THR324, ASN81, CYS83, GLU322, HIS85, PRO284, VAL283, SER282, GLY281, OTR410, and HIS244	ASN81, CYS83, HIS85, and HIS244

Tanshinone IIA	VAL248, HIS244, OTR410, VAL283, SER282, PRO277, PHE264, and ARG268	VAL283 and SER282

Lithospermic acid	PRO270, THR261, GLY281, ARG268, PHE264, ASN260, MET257, SER282, VAL283, OTR410, VAL248, and HIS244	GLY281, ARG268, PHE264, SER282, and VAL283

Salvianolic acid A	ASN81, CYS83, HIS85, VAL283, SER282, OTR410, HIS244, VAL248, PHE264, and ARG268	CYS83, HIS85, VAL283, SER282, and HIS244

Caffeic acid	PHE264, ARG268, OTR410, GLY281, VAL283, and SER282	PHE264, ARG268, VAL283, and SER282

Danshensu	PHE264, ARG268, OTR410, GLY281, VAL283, and SER282	ARG268, VAL283, and SER282

Phenylalanine	HIS244, HIS85, THR84, CYS83, GLU322, VAL283, and ASN81	HIS85, THR84, and GLU322

Rosmarinic acid	ARG268, PHE264, VAL248, HIS244, GLU322, OTR410, HIS85, SER282, and VAL283	ARG268, GLU322, SER282, and VAL283

Salvianolic acid E	ARG268, PHE264, VAL248, HIS244, HIS85, SER282, VAL283, OTR410, and PRO284	ARG268, SER282, VAL283, and HIS244

Anhydrosafflor yellow B	GLU322, HIS244, HIS85, VAL248, VAL283, PRO284, ASN81, OTR410, ASN260, SER282, GLY281, PHE264, THR261, ARG268, and PRO277	GLY281 and ARG268

6-Hydroxykaempferol-3,6,7-*O*-*β*-D-glucoside	THR324, GLU322, ASN81, HIS85, HIS244, OTR410, VAL283, PRO284, GLY281, and SER282	GLU322, ASN81, HIS85, and HIS244

6-Hydroxykaempferol 3-*O*-rutinoside-6-*O*-glucoside	THR324, ASN81, HIS85, OTR410, VAL283, PRO284, GLY281, and SER282	ASN81, HIS85, and GLY281

## Data Availability

The data used to support the findings of this study are available from the corresponding author upon request.
